# How does disclosure of the HIV positive status modify drug use?

**DOI:** 10.1186/1471-2334-13-S1-P18

**Published:** 2013-12-16

**Authors:** Adrian Octavian Abagiu, Florin Matache Dună, Elena Daniela Bunescu, Ioana Cristina Fierbințeanu, Dan Popescu, Rafael Ianoş-Rancovici, Anca Gubăr, Poliana Ortansa Radu

**Affiliations:** 1 National Institute for Infectious Diseases “Prof. Dr. Matei Balş”, Bucharest, Romania; 2 Romanian Anti-AIDS Association (ARAS), Bucharest, Romania

## Background

We wanted to examine how disclosure of the HIV positive status modifies risky behaviors and drug use in patients in substitution at the ARENA center from the National Institute for Infectious Diseases “Prof. Dr. Matei Balş”, Bucharest.

## Methods

The prospective study enrolled 94 patients diagnosed with HIV after IV consumption of “legal highs” and being in oral substitution treatment since March 2012. The final analysis included 71 patients who were in treatment in the range of 15 months from the initiation of the study and answered at least 2 of the quarterly surveys. All patients had urine tests done monthly.

## Results

13 of the patients (18.3%) were women; the average age was 31.2 years with an average IV drug use of 8.7 years, of which 19 months with legal highs also. The average number of relapses per patient prior to the study period was 4.7, due to lack of participation to psychotherapy. Only 17 patients (24%) managed to quit legal highs consumption in the studied period. 15 patients (21%) were out of therapy for a period of over 21 days within the study period. Most of them are with low literacy (6.2 grades), 91% were incarcerated. Of the 24 patients in antiretroviral treatment, 3 dropped out of therapy. Only 7 patients had positive urine test for opiates, but other 47 had recognized legal highs intermittent consumption.

## Conclusion

Paradoxically, disclosing the HIV-positive status, with all the counseling and even involving free access to substitution therapy, caused relapse in the majority of patients. The main idea is that if they are going to die, at least to die “drugged and happy”. On the other hand this signals the need for new approaches in counseling.

